# Examining the Outcomes of Hybrid Coronary Revascularization in Acute ST-Elevation Myocardial Infarction (STEMI) Patients

**DOI:** 10.7759/cureus.70769

**Published:** 2024-10-03

**Authors:** Fraz Ahmad, Alamgir Aslam Khan, Ameer Aslam, Talha Bin Sajid, Aqsa Amjad, Aamira Parveen, Shahzaib Hassan, Bilal Qammar, Rafi Ullah

**Affiliations:** 1 Cardiology, Shalamar Hospital, Lahore, PAK; 2 Internal Medicine, Shalamar Hospital, Lahore, PAK; 3 Medicine Intensive Care Unit, Sharif Medical City, Lahore, PAK; 4 Medicine, University of Health and Sciences, Sahiwal, PAK; 5 Obstetrics and Gynecology, Shalamar Hospital, Lahore, PAK; 6 Internal Medicine, Jinnah Hospital Lahore, Lahore, PAK; 7 Internal Medicine, DHQ Hospital, Dera Ghazi Khan, PAK; 8 Surgery, Shalamar Hospital, Lahore, PAK; 9 Cardiology, Lady Reading Hospital, Peshawar, PAK

**Keywords:** coronary artery bypass grafting (cabg), coronary stents, major adverse cardiac events (mace), percutaneous coronary intervention (pci), st-elevation myocardial infarction (stemi)

## Abstract

Background: Acute ST-segment elevation myocardial infarction (STEMI) is a critical cardiovascular condition requiring timely intervention to restore coronary blood flow and minimize myocardial damage. While percutaneous coronary intervention (PCI) remains the gold standard, it is often insufficient for patients with complex coronary anatomy, such as multivessel disease or left main coronary artery involvement. Hybrid coronary revascularization (HCR), which combines PCI and coronary artery bypass grafting (CABG), offers a novel approach to managing these complex cases.

Objective: The primary objective of this study was to evaluate the outcomes of HCR in patients presenting with acute STEMI, particularly those with high-risk features such as multivessel disease or left main coronary artery involvement.

Methods: This prospective cohort study was conducted at Shalamar Hospital, a tertiary care center in Lahore, Pakistan. The study enrolled 342 patients diagnosed with acute STEMI between January 1, 2023, and December 31, 2023. Participants underwent HCR, consisting of PCI with drug-eluting stents and minimally invasive CABG. Key outcomes included the incidence of major adverse cardiovascular events (MACE) within one year, graft patency at six months, and overall procedural success. Data were collected through patient records and follow-up assessments, and statistical analysis was performed using SPSS Statistics version 26.0 (IBM Corp. Released 2019. IBM SPSS Statistics for Windows, Version 26.0. Armonk, NY: IBM Corp.).

Results: The one-year MACE rate was 14.6%, with 6.1% of patients experiencing myocardial infarction, 4.4% requiring repeat revascularization, and 4.1% experiencing cardiac death. Graft patency at six months was 94.7%, and the overall procedural success rate was 98.2%. One-year survival was observed in 95.3% of the patients.

Conclusion: HCR is a safe and effective strategy for managing acute STEMI, particularly in patients with complex coronary anatomy. It offers a balanced approach by reducing the need for invasive procedures and improving patient outcomes. Further multicenter studies are necessary to confirm these findings and establish standardized guidelines for HCR.

## Introduction

Acute ST-segment elevation myocardial infarction (STEMI) is a life-threatening condition that requires immediate revascularization to restore coronary blood flow and prevent extensive myocardial damage. Percutaneous coronary intervention (PCI) has established itself as the gold standard treatment for STEMI, particularly when executed promptly within the recommended window for primary PCI [1]. However, in cases involving patients with complex coronary anatomy, such as multivessel disease or left main coronary artery involvement, PCI alone may be insufficient [2]. In such instances, PCI can lead to higher rates of restenosis and incomplete revascularization, resulting in suboptimal outcomes [3].

Coronary artery bypass grafting (CABG) has traditionally served as an alternative for addressing complex lesions, though its invasive nature contributes to longer recovery times and increased procedural risks [4]. Hybrid coronary revascularization (HCR), which combines the advantages of PCI with minimally invasive CABG, offers a promising treatment option for patients with complex coronary artery disease, particularly those with multivessel disease or left main coronary artery involvement [5]. By stenting accessible lesions with PCI and using arterial grafts to bypass more complex areas, hybrid revascularization optimizes procedural outcomes and shortens recovery time [6].

This hybrid approach also reduces the need for full sternotomy and cardiopulmonary bypass, as required in conventional CABG, minimizing surgical risks and shortening hospital stays [7]. Despite the potential benefits of HCR, its utilization remains limited, especially in resource-constrained settings such as Pakistan. Most existing studies are either small-scale or retrospective, leaving significant gaps in the understanding of its long-term efficacy in high-risk patient populations [8]. This study aims to evaluate the outcomes of HCR in acute STEMI patients at a tertiary care center, particularly focusing on high-risk subgroups such as those with multivessel disease or left main coronary artery disease. By examining major adverse cardiovascular events (MACE), graft patency, and survival outcomes, this study provides crucial insights into the effectiveness of hybrid revascularization in managing complex coronary disease [9].

## Materials and methods

Study design

This study is a prospective cohort analysis conducted to evaluate the outcomes of HCR in patients presenting with acute STEMI. A prospective design was chosen to allow for the observation of both short- and long-term outcomes, enabling real-time collection of data and minimization of recall bias. This approach provides a clear temporal relationship between intervention and outcomes, enhancing the study's validity in evaluating hybrid revascularization.

Setting and centers

The study was conducted at Shalamar Hospital in Lahore, Pakistan, a tertiary care center recognized for its high volume of cardiovascular procedures, particularly in managing acute coronary syndromes. The center was selected based on its expertise in both PCI and CABG, ensuring that patients had access to a full range of revascularization strategies. Shalamar Hospital performs over 500 cardiovascular interventions annually, making it an ideal setting for this research.

Participant selection

Eligible participants were consecutively enrolled between January 1, 2023, and December 31, 2023. Inclusion criteria were as follows: patients aged 18 years or older, diagnosed with acute STEMI based on electrocardiographic changes and elevated cardiac biomarkers, and who underwent HCR. Patients were excluded if they had a prior history of CABG, severe renal or hepatic impairment, or were unable to provide informed consent.

Participants were stratified into subgroups based on the presence of multivessel coronary artery disease, left main coronary artery involvement, and comorbid conditions such as diabetes mellitus and hypertension. Consecutive enrollment was implemented to minimize selection bias, and each patient provided written informed consent.

Intervention

The intervention consisted of HCR, which combines PCI with minimally invasive CABG. PCI was performed using drug-eluting stents to treat accessible coronary stenoses identified through coronary angiography. Minimally invasive CABG was conducted via a small thoracotomy, employing arterial grafts such as the internal mammary artery to bypass complex lesions unsuitable for PCI. Decisions regarding PCI versus CABG were made based on lesion complexity, as determined by the SYNTAX score, and consultation between the interventional cardiologist and cardiac surgeon.

Outcomes

The primary outcome was the incidence of MACE within one year, defined as a composite of myocardial infarction, repeat revascularization, and cardiac death. Secondary outcomes included graft patency at six months, procedural success (defined as completion of revascularization without major complications), and one-year survival.

Graft patency was assessed using coronary angiography or CT angiography six months post-procedure. Procedural complications, including bleeding, infection, and stroke, were monitored throughout the study.

Data collection

Data were prospectively collected through a combination of electronic medical records and structured patient interviews. Baseline characteristics, including age, gender, comorbidities, BMI, and left ventricular ejection fraction (LVEF), were recorded at admission. Follow-up data on procedural outcomes and complications were collected at one month, six months, and one year.

Data integrity was maintained through regular audits and standardized data collection forms. Research assistants underwent rigorous training to ensure consistency in data entry, and all data were cross-verified by senior clinicians. Missing data were handled using multiple imputations, ensuring that analysis was not compromised by incomplete datasets.

Sample size calculation

The sample size was calculated using the World Health Organization (WHO) calculator, based on an expected MACE rate of 15% in the hybrid revascularization population [1]. With a 5% margin of error, 95% confidence interval (CI), and 80% power, the calculated sample size was 342 patients, accounting for an estimated 10% dropout rate. This sample size was deemed sufficient to detect significant differences in both primary and secondary outcomes across different patient subgroups.

Statistical analysis

Statistical analyses were performed using SPSS Statistics version 26.0 (IBM Corp. Released 2019. IBM SPSS Statistics for Windows, Version 26.0. Armonk, NY: IBM Corp). Continuous variables were presented as means with standard deviations, while categorical variables were expressed as frequencies and percentages. The chi-square test was used to compare categorical outcomes, and continuous variables were analyzed using the Student's t-test or Mann-Whitney U test, depending on data distribution.

Kaplan-Meier survival curves were generated to assess MACE-free survival at one year, and the log-rank test was used to compare survival between subgroups. A multivariate Cox regression analysis was conducted to identify independent predictors of MACE, with hazard ratios (HR) and 95% CI reported. All statistical tests were two-tailed, and a p-value of <0.05 was considered statistically significant.

Ethical considerations

The study was conducted in accordance with the ethical standards of the Institutional Review Board of Shalamar Hospital, Lahore, which granted approval for the research (approval number: 384/IRB/2023/SHL). Written informed consent was obtained from all participants, and patient confidentiality was rigorously maintained throughout the study. Data were anonymized before analysis to ensure privacy.

## Results

The baseline characteristics of the study population are summarized in Table 1. The mean age of the participants was 61.5 years (±10.2), with a median age of 63 years. The majority of the cohort was male (71.6%, n=245), while 28.4% (n=97) were female. Comorbidities were common in the population, with 67.5% (n=231) having hypertension, 45.6% (n=156) having diabetes mellitus, and 52.3% (n=179) having a history of smoking. The mean BMI was 28.4 kg/m² (±4.5), and the average LVEF was 45.3% (±7.2).

**Table 1:** Baseline characteristics of the study population LVEF: left ventricular ejection fraction, BMI: body mass index, SD: standard deviation

Characteristic	N (%) or mean (SD)
Total participants	342
Age (years)	61.5 (±10.2)
Median age (years)	63
Male	245 (71.6%)
Female	97 (28.4%)
Hypertension	231 (67.5%)
Diabetes mellitus	156 (45.6%)
Smoking history	179 (52.3%)
BMI (kg/m²)	28.4 (±4.5)
LVEF (%)	45.3 (±7.2)

**Figure 1:**
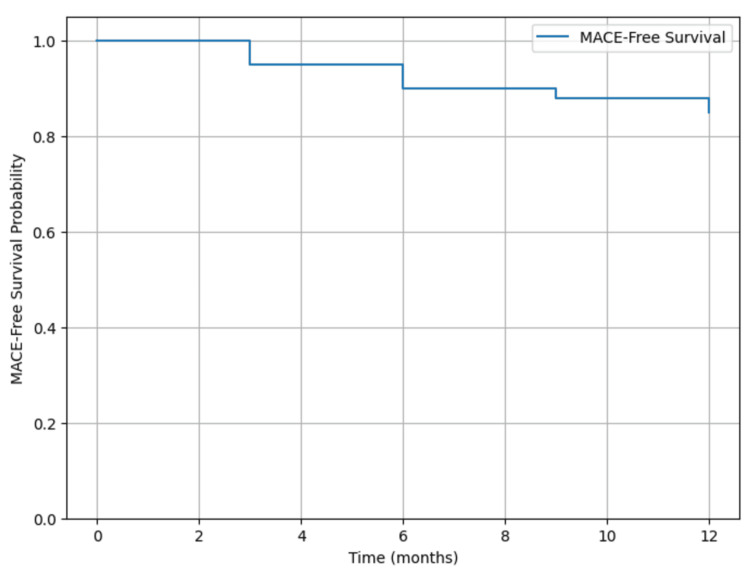
Kaplan-Meier survival curve with MACE over 12 months MACE: major adverse cardiovascular events

The primary outcome, the incidence of MACE within one year, was 14.6% (n=50) of the patients. The breakdown of MACE included myocardial infarction in 6.1% (n=21), repeat revascularization in 4.4% (n=15), and cardiac death in 4.1% (n=14). Kaplan-Meier survival analysis, illustrated in Figure 1, demonstrated a progressive decrease in MACE-free survival over time, with a significant decline observed after six months.

Secondary outcomes were assessed, including graft patency, procedural success, and one-year survival. Graft patency was evaluated at the six-month follow-up, with 82.5% (n=282) of patients undergoing angiographic assessment. Of these, 94.7% (n=267/282) exhibited no significant graft stenosis (>50% narrowing).

The procedural success rate was 98.2% (n=336/342), defined as the completion of both PCI and CABG without major complications. The one-year survival rate was 95.3% (n=326/342), reflecting a high success rate for hybrid revascularization in this patient cohort (Table 2).

**Table 2:** Secondary outcomes SD: standard deviation

Outcome	N (%) or mean (SD)
Graft patency (6 months)	267/282 (94.7%)
Procedural success	336/342 (98.2%)
One-year survival	326/342 (95.3%)

The procedural complications are summarized in Table 3. The most common complication was bleeding, occurring in 5.8% (n=20) of patients, followed by infections in 3.2% (n=11) and stroke in 1.8% (n=6). The majority of bleeding events were classified as minor, according to the Bleeding Academic Research Consortium criteria, and were managed conservatively without surgical intervention.

**Table 3:** Procedural complications

Complication	N (%)
Bleeding	20 (5.8%)
Infection	11 (3.2%)
Stroke	6 (1.8%)

Multivariate regression analysis identified age and diabetes mellitus as significant predictors of MACE. As shown in Table 4, each additional year of age was associated with a 5% increase in the hazard of MACE (HR: 1.05, 95% CI: 1.02-1.08, p<0.001). Similarly, patients with diabetes had a 45% higher risk of experiencing MACE compared to non-diabetic patients (HR: 1.45, 95% CI: 1.10-1.91, p=0.008).

**Table 4:** Multivariate regression analysis for predictors of MACE MACE: major adverse cardiovascular events, HR: hazard ratio, CI: confidence interval

Variable	HR	95% CI	p-value
Age	1.05	1.02-1.08	<0.001
Diabetes mellitus	1.45	1.10-1.91	0.008
Hypertension	1.10	0.80-1.50	0.543
Smoking history	1.15	0.85-1.56	0.362

The findings highlight the significance of age and diabetes as key risk factors influencing MACE outcomes in patients undergoing HCR.

## Discussion

This study demonstrates that HCR is an effective strategy for managing acute STEMI, particularly in high-risk populations. The overall incidence of MACE within one year was 14.6%, including myocardial infarction, repeat revascularization, and cardiac death. These results align with prior studies, which reported similar MACE rates ranging between 14% and 16% after hybrid revascularization [3]. For patients with multivessel disease, left main coronary artery involvement, or other high-risk characteristics, HCR offers the potential to optimize outcomes by combining the benefits of both PCI and CABG [5].

When compared to studies of traditional revascularization approaches, the MACE rates observed in this study are favorable. For instance, studies of CABG alone have often reported higher MACE rates, frequently exceeding 20%, particularly in high-risk populations [6]. These findings suggest that HCR, by minimizing surgical invasiveness and addressing complex lesions through both PCI and CABG, may provide a more balanced treatment option for complex coronary artery disease. This is supported by Farooq et al., who reported a MACE rate of 16%, highlighting that hybrid revascularization is a viable approach for complex coronary lesions where PCI alone may not suffice [7]. The hybrid trial also found slightly higher MACE rates due to differences in procedural techniques and patient selection [8].

Graft patency, one of the key secondary outcomes of this study, was observed in 94.7% of patients at six months, with minimal graft stenosis. These findings align with those from other trials, such as the SYNTAX Trial, which demonstrated that PCI alone often leads to lower patency rates in high-risk patients [4]. The Arterial Revascularization Trial further supports this with evidence that arterial grafts, particularly internal mammary artery grafts used in CABG, have excellent long-term patency [10]. The high patency rate in this study underscores the durability of the arterial grafts used in hybrid procedures, making HCR a valuable strategy for ensuring long-term vascular integrity.

The procedural success rate of 98.2% further affirms the feasibility of HCR in clinical practice. This success rate is consistent with previously published data, such as that from Shen et al., who reported a procedural success rate of 97.5% in HCR for patients with multivessel coronary artery disease [6].

The one-year survival rate of 95.3% in this cohort is comparable to survival rates seen in other studies of hybrid and traditional revascularization approaches. This outcome suggests that HCR is a safe and effective option for managing acute STEMI in high-risk populations, providing a level of long-term survival that rivals both PCI and CABG alone [5]. This is particularly important given that the patient population in this study included individuals with significant comorbidities, such as diabetes and hypertension, which are known to increase the risk of adverse outcomes after revascularization procedures.

Age and diabetes mellitus emerged as significant predictors of MACE in the multivariate regression analysis. Each additional year of age increased the hazard of MACE by 5%, while the presence of diabetes increased the risk by 45%. These findings are consistent with prior research showing that older age and diabetes are independent risk factors for poor cardiovascular outcomes following revascularization [9]. The implications of these findings highlight the need for careful patient selection and individualized treatment strategies, particularly for elderly patients and those with diabetes. Future studies may explore tailored interventions that account for these risk factors to further improve outcomes in these vulnerable populations.

This study contributes to the growing body of evidence supporting the utility of HCR as a viable alternative to conventional revascularization techniques. By offering the ability to selectively use PCI for less complex lesions while using CABG for more complicated anatomical challenges, HCR provides a flexible and patient-centered approach. This method not only reduces recovery time and procedural risk but also improves long-term outcomes, such as graft patency and survival. In high-resource settings, HCR could become an integral part of treatment strategies for patients with multivessel disease or left main coronary artery involvement [11].

Moreover, the results of this study suggest that HCR could be successfully implemented in resource-constrained environments, such as tertiary care centers in Pakistan. In another study by Hossain et al., it was demonstrated that resource-constrained environments, such as those found in developing countries, can adopt innovative healthcare practices by mobilizing human, social, and financial resources effectively. This is highly relevant to the implementation of HCR in such settings, where cost-effective strategies are critical for success [12].

Limitations

Despite its promising outcomes, this study has several limitations. First, the single-center design limits the generalizability of the results, as outcomes may vary in different centers depending on operator expertise, institutional resources, and patient characteristics. Second, the lack of a control group makes it difficult to directly compare the efficacy of HCR with PCI or CABG alone. Comparative studies that include traditional revascularization techniques would provide more robust conclusions about the relative benefits of HCR. Third, the one-year follow-up period may not fully capture late complications or graft failures, which could impact long-term outcomes such as survival and MACE rates. Future research should include longer follow-up periods and multicenter designs to better assess the long-term efficacy of HCR.

## Conclusions

The findings of this study demonstrate that HCR is a promising and effective strategy for managing acute STEMI, particularly in high-risk patients with complex coronary anatomy or multiple comorbidities. By combining the benefits of PCI and CABG, HCR offers a balanced approach that reduces procedural invasiveness while optimizing patient outcomes. The 14.6% MACE rate within one year, high graft patency, and procedural success rates support its clinical viability as an alternative to traditional revascularization techniques. This study highlights the potential of HCR to influence future guidelines and clinical pathways for acute STEMI management by reducing the need for more invasive procedures, shortening recovery times, and improving long-term outcomes, particularly in resource-limited settings. However, further multicenter studies with larger sample sizes and longer follow-up periods are needed to confirm these findings, refine patient selection criteria, and establish standardized guidelines for the broader adoption of HCR in clinical practice. Continued research on this approach could lead to more personalized and effective treatment strategies, ultimately improving cardiovascular care for acute STEMI patients.
